# The future at risk: Tackling newborn and child mortality amidst a global health crisis

**DOI:** 10.1371/journal.pgph.0004519

**Published:** 2025-04-23

**Authors:** Etienne V. Langlois, Giulia Gasparri, Rajat Khosla

**Affiliations:** Partnership for Maternal, Newborn and Child Health (PMNCH), World Health Organization (WHO), Geneva, Switzerland; PLOS: Public Library of Science, UNITED STATES OF AMERICA

The world is facing unprecedented threats to global health and international development. The decrease in international financing for health, the rising geopolitical tensions and threats to multilateralism, the increasing number of conflicts, and the intensifying threats of climate change all pose a growing concern for the health and well-being of the most vulnerable populations. Newborns and children are always the most affected by these compounding crises, which risk washing away decades of progress in newborn and child health.

The new estimates from the UN-IGME report reveal that in 2023, approximately 4.8 million children died before turning 5 [1]. Each minute, death comes to more than 9 children under the age of five – for reasons that could be largely avoided. Newborns accounted for 2.3 million of these deaths - 48% of the annual total [1]. In 2023, almost 2 million babies were stillborn at 28 weeks or more of gestation – or the last trimester of pregnancy [1].

**Fig 1:**
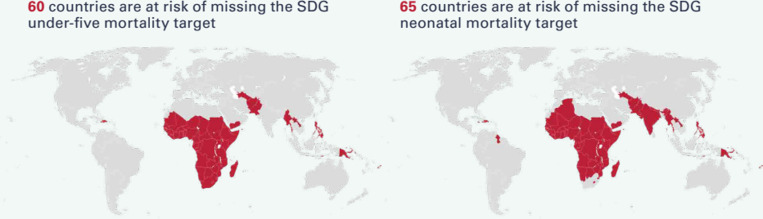
Countries at risk of missing SDG 3.2.1 and 3.2.2 [ 1].

In addition, the decline in newborn mortality rates and under-five mortality rates has slowed down during the Sustainable Development Goal (SDG) era [1]. An increasing number of countries are witnessing a stagnation of the reduction, with the overall decline in neonatal mortality being slower than that of post-neonatal under-5 mortality. With these current trends, about 60 countries are at risk of missing the SDG target for child mortality (3.2.1) and 65 countries will miss the newborn mortality target (3.2.2) (Fig 1) [1]. If these countries meet the SDG targets by 2030, 8 million more children would live through their fifth birthday [1].

These worrying numbers are associated with another unacceptable truth: where you are born determines your chances of survival. In the highest-mortality country, the risk of death before age 5 is 80 times that of the lowest-mortality country [1]. Moreover, the risk of mortality before the age of five is particularly high in humanitarian and fragile settings, including conflicts. Children born in countries classified as “fragile and conflict-affected settings” (FCS) face nearly three times the risk of mortality before the age of five compared to those born in non-FCS countries [1].

The reality is that many of these deaths are preventable. We know the causes, and we know the solutions. Since 2000, the global under-five mortality rate has declined by 52%, from 77 deaths per 1,000 live births in 2000 to 37 in 2023 [1]. For the first time, global child mortality figures for children under five years fell under the five-million mark in 2022 [2]. Yet, the overall numbers of deaths amongst newborns and children are still highly unacceptable.

Preterm birth is the leading cause of death of children under-five, responsible for 1 in 5 deaths [3]. The chances of surviving from preterm birth are incredibly unjust depending on where you are born. 9 in 10 extremely preterm babies (<28 weeks) survive in high-income countries, compared to fewer than 1 in 10 survive in low-income countries [3]. Other major causes of child mortality are: pneumonia, birth asphyxia and trauma, malaria, and diarrhea. Additionally, congenital anomalies are a major killer of children under-five, including congenital heart and neural tube defects. Moreover, malnutrition exacerbates the threats to child mortality, as children with severe acute malnutrition are more likely to die from common childhood illnesses [4].

We must act now and advance the implementation of evidence-based and cost-effective solutions. This includes upholding essential life-saving interventions and access to commodities. Investing in newborn and child health services is not only a moral priority, but also a good financial investment. The *Global Health 2050* report highlights that prioritizing investments in routine childhood immunization, treatment of acute child illness, pregnancy and childbirth services, and family planning are key cost-effective interventions to reduce premature mortality [5]. Investments in integrated reproductive, maternal, newborn and child health packages would return between US$9–20 for every US$1 spent [6].

Beyond mortality reduction, investing in children’s health will address morbidity and improve child and adolescent well-being, yielding key benefits across the lifecourse [7]. It is therefore imperative for countries to scale up domestic investments in newborn and child health through primary health care and universal health coverage approaches.The movement towards stronger domestic financing of essential maternal, newborn, and child health (MNCH) services should be informed by the Lusaka Agenda, providing a foundation for the sustainability of domestically financed health systems [8]. This will help to address inequities and out-of-pocket payments for newborn and child health services. Direct health expenditures by households accounted for a third (32.9%) of total health expenditure in Africa in 2021 [9].

Moreover, progress cannot be achieved in newborn and child health without focusing on the areas with the highest burden of mortality, namely fragile and conflict-affected settings. There is an urgent need to support equity-enhancing efforts focused on regions with the highest mortality and morbidity including, sub-Saharan Africa and South Asia. This requires the explicit prioritization of newborn and child health in emergency responses, early warning systems and disaster reduction plans including those related to climate change, pandemic prevention, preparedness and response, and peace building efforts.

Most importantly, to achieve progress, we need political will. The 77^th^ World Health Assembly resolution, “Accelerating progress towards reducing maternal, newborn, and child mortality to achieve Sustainable Development Goals 3.1 and 3.2” [10], demonstrated Member States’ renewed willingness to prioritize this agenda. We now need to move from promises to implementation. The resolution calls Member States to scale up cost-effective interventions in MNCH services, including routine immunization programmes and family planning, in national health strategies, by ensuring sufficient human resources for health and prioritizing preventable mortality and morbidity national health plans. Moreover, countries must integrate high-impact interventions and commodities addressing the key causes of mortality, including the prevention and care for preterm births, and essential immunization. We must strive to reach “zero-dose” children, which constitute around 14.5 million children every year who do not receive basic vaccines [11].

All stakeholders have a role to play in advocating for the prioritization of this agenda and the implementation of the commitments made to ensure no newborn and child is left behind. Thanks to the improvements in child mortality rates, the world saved 94.8 million children since 2000 [1]. Faster progress is possible and within our reach. We must acknowledge this crisis as a global emergency and act now to safeguard our children and secure our future.
